# Dendritic Inhibition Terminates Plateau Potentials in CA1 Pyramidal Neurons

**DOI:** 10.1523/JNEUROSCI.1540-25.2026

**Published:** 2026-04-17

**Authors:** Lee O. Vaasjo, Shawn E. Kotermanski, Tiya Patel, Hengyue J. Shi, Robert Machold, Simon Chamberland

**Affiliations:** ^1^Department of Neuroscience, Center for Neuroscience, University of Pittsburgh, Pittsburgh, Pennsylvania 15260; ^2^Neuroscience Institute, New York University Grossman School of Medicine, New York, New York 10016

**Keywords:** dendrites, hippocampus, inhibition, interneurons, plateau potentials

## Abstract

In CA1 pyramidal neurons (CA1-PYRs), plateau potentials control synaptic plasticity and the emergence of place cell identity. Here, we show that dendritic inhibition terminates plateaus in an all-or-none manner in CA1-PYRs recorded in acute hippocampal slices from mice of either sex. Plateaus were initially resistant to inhibition but became increasingly susceptible to termination as they progressed. Two subtypes of dendrite-targeting oriens-lacunosum moleculare (OLM) interneurons, accessed in transgenic mice based on the expression of the genes *Ndnf* or *Chrna2* (OLM^Ndnf^ and OLM^α2^, respectively), could terminate plateau potentials. OLM^Ndnf^ generated slower postsynaptic currents that terminated plateaus more effectively than OLM^α2^. Voltage-gated Ca^2+^ channels (VGCCs) were necessary for plateaus, which were prolonged by blocking small-conductance Ca^2+^–activated K^+^ channels (SK). A single-compartment model with these two conductances recapitulated core experimental findings and provided a mechanistic explanation for terminations. Plateaus arose from VGCCs maintained in the active state by sustained Ca^2+^ influx, a positive feedback loop that was quasi-balanced by I_SK_. Inhibition terminated plateaus by driving the membrane potential below a dynamic threshold to deactivate VGCCs and end the positive feedback loop. Similar all-or-none termination dynamics were observed for plateaus evoked under cholinergic modulation. Lastly, two-photon Ca^2+^ imaging showed that plateaus evoke large dendritic Ca^2+^ transients that were graded by terminations. Overall, our results demonstrate how the feedback inhibitory circuit interacts with intrinsic cellular mechanisms to regulate plateau potentials and shape dendritic Ca^2+^ signals in CA1-PYRs.

## Significance Statement

Plateau potentials are critical biophysical events that drive memory-related synaptic plasticity in the hippocampus, yet their underlying regulatory mechanisms remain incompletely understood. Here, we reveal that synaptic inhibition can abruptly terminate plateaus in CA1 pyramidal neurons. This all-or-none termination results from a nonlinear interaction between voltage-gated Ca^2+^ channels and SK channels. Using intersectional genetics, we identify two dendrite-targeting interneuron subtypes that differentially modulate plateau duration. Two-photon Ca^2+^ imaging further shows that plateau termination converts these binary events into graded dendritic Ca^2+^ signals. Overall, these results demonstrate that feedback inhibition regulates the duration of plateaus, adding a critical layer of control over dendritic computation.

## Introduction

Neurons actively integrate information in their dendrites where multiple forms of dendritic spikes amplify synaptic potentials. Plateau potentials are a form of dendritic spikes characterized by prolonged membrane depolarization. In hippocampal CA1 pyramidal cells (CA1-PYRs), plateaus evoked in single dendritic branches can last for hundreds of milliseconds and contribute to multiple forms of synaptic plasticity (Wei et al., 2001; Cai et al., 2004; Takahashi and Magee, 2009; Zhao et al., 2022). In behavioral timescale plasticity (BTSP), a form of one-shot learning, a single plateau suffices to strengthen excitatory synaptic inputs to enable place field formation in CA1-PYRs (Bittner et al., 2017; Magee, 2026). Therefore, plateaus are a biophysical signal controlling synaptic plasticity and memory.

Inhibition and membrane hyperpolarization interfere with multiple forms of dendritic spikes including plateaus (Russell and Hartline, 1978; Hounsgaard and Mintz, 1988; Callaway et al., 1995; Kim et al., 1995; Buzsaki et al., 1996; Miles et al., 1996; Booth et al., 1997; Dicaprio, 1997; Milstein et al., 2015; Bloss et al., 2016; Doron et al., 2017; Du et al., 2017). Plateaus represent a depolarized state with elevated driving force for GABA_A_R-mediated Cl^−^ currents. In CA1-PYRs, plateaus are generated and maintained by multiple conductances, likely much smaller than those supporting action potentials (APs), potentially rendering plateaus vulnerable to synaptic activity (Fraser and MacVicar, 1996; Wei et al., 2001; Cai et al., 2004; Takahashi and Magee, 2009; Hsu et al., 2018). How inhibition controls ongoing plateaus in CA1-PYRs remains poorly understood.

A heterogeneous population of GABAergic interneurons (INs) controls activity across specific spatial domains of CA1-PYRs (Freund and Buzsaki, 1996; Miles et al., 1996; Bloss et al., 2016; Pelkey et al., 2017; Booker and Vida, 2018). Among these, somatostatin-expressing INs (*Sst*-INs) provide key feedback inhibition to CA1-PYRs dendrites (Lacaille et al., 1987; Maccaferri and McBain, 1995; Wei et al., 2001; Losonczy et al., 2002; Cai et al., 2004; Pouille and Scanziani, 2004), shaping local synaptic input integration (Leao et al., 2012; Lovett-Barron et al., 2012; Muller et al., 2012; Udakis et al., 2020), burst firing (Royer et al., 2012), and hippocampal-dependent memory (Lovett-Barron et al., 2014; Artinian et al., 2019; Honore et al., 2021; Udakis et al., 2025). Single-cell transcriptomics and intersectional genetic tools have recently confirmed extensive heterogeneity within *Sst*-INs and enabled access to distinct subtypes (Harris et al., 2018; Chamberland et al., 2024). We recently identified that neurons coexpressing *Ndnf* and *Nkx2-1* adopt an oriens-lacunosum moleculare (OLM) identity (OLM^Ndnf^) and preferentially innervate CA1-PYRs over fast-spiking INs, whereas *Chrna2*-expressing OLMs (OLM^α2^) strongly innervate both targets (Leao et al., 2012; Chamberland et al., 2024). How subtypes of OLMs control CA1-PYRs dendritic activity remain unknown.

Here, we discovered that dendritic inhibition terminates plateaus in an all-or-none manner in CA1-PYRs. Plateaus were initially resistant to inhibitory termination but became increasingly susceptible as they progressed over time. Optogenetic stimulation of OLM^Ndnf^ terminated plateaus more effectively than OLM^α2^, an effect attributed to the slower synaptic currents mediated by OLM^Ndnf^. Experiments and biophysical modeling revealed the mechanisms governing plateau termination, highlighting that synaptic inhibition must overcome rebalancing of intrinsic conductances to drive *V*_M_ below a dynamic threshold. Finally, two-photon Ca^2+^ imaging revealed that plateau termination effectively grades dendritic Ca^2+^ elevations.

## Materials and Methods

### Animals

All experiments on animals were approved by IACUC at the University of Pittsburgh. Animals were given access to food and water *ad libitum*. Experiments were performed on mice of either sex. Animals used for experiments were between Postnatal Day (P)25 and P125. The following transgenic animals were used in this study: *Sst-Cre;;Ai32*, *Chrna2-Cre;;Ai9*, *Ndnf-FlpO;;Nkx2-1-Cre;;Ai80*, *Chrna2-Cre;;Sst-FlpO;;Ai80*, *Ndnf-FlpO;;Nkx2-1-Cre;;Ai65*, and *Ndnf-FlpO;;Chrna2-Cre;;Ai224*. Littermates from these crosses were also used in experiments. The animals were obtained by breeding the following animals: *Sst-Cre* (Sst^tm2.1(cre)Zjh^/J; JAX: 013044) were maintained as homozygous (Taniguchi et al., 2011); *Sst-FlpO* (B6J.Cg-Sst^tm3.1(flpo)Zjh^/AreckJ; JAX: 031629) were maintained as homozygous (He et al., 2016); *Chrna2-Cre* were maintained as hemizygous (gift from Dr. Klas Kullander; Leao et al., 2012). *Ndnf-FlpO* were maintained as heterozygous (Dr. Robert Machold, NYU; Chamberland et al., 2024). *Nkx2-1-Cre* [C57BL/6J-Tg(Nkx2-1-cre)2Sand/J; JAX: 008661] were maintained as hemizygous (Xu et al., 2008). *Ndnf-FlpO;;Nkx2-1-Cre* were maintained as het/hemi. *Chrna2-Cre;;Sst-FlpO* were maintained as hemi/hom. The following reporter lines were used and maintained as homozygous:

Ai9 [B6.Cg-Gt(ROSA)26Sor^tm9(CAG-tdTomato)Hze^/J; JAX: 007909; Madisen et al., 2010]
Ai32 [B6.Cg-Gt(ROSA)26Sor^tm32(CAG-COP4*H134R/EYFP)Hze^/J; JAX: 024109; Madisen et al., 2012]
Ai65 [B6;129S-Gt(ROSA)26Sor^tm65.1(CAG-tdTomato)Hze^/J; JAX: 021875; Madisen et al., 2015]
Ai80 [B6.Cg-Gt(ROSA)26Sor^tm80.1(CAG-COP4*L132C/EYFP)Hze^/J; JAX: 025109; Daigle et al., 2018]
Ai224 [B6.Cg-Igs7^tm224(CAG-EGFP,CAG-dTomato)Tasic^/J; JAX: 037382; Ben-Simon et al., 2024]

### Acute hippocampal slice preparation

Animals were deeply anesthetized with isoflurane using the drop-jar method before decapitation. The brain was rapidly extracted and immersed in oxygenated (95% O_2_, 5% CO_2_) ice-cold sucrose–based artificial cerebrospinal fluid (sucrose aCSF) containing the following (in mM): 185 sucrose, 25 NaHCO_3_, 2.5 KCl, 25 glucose, 1.25 NaH_2_PO_4_, 10 MgCl_2_, and 0.5 CaCl_2_, pH 7.4, 300 mOsm. Brain hemispheres were glued on a specimen disk. Acute slices (300 µm) were prepared on a vibratome (VT1200 S, Leica), and the slicing chamber was maintained on ice and continuously oxygenated. Slices were allowed to recover in oxygenated and heated (32°C) sucrose aCSF for 30 min, before being transferred to oxygenated and heated (32°C) recording aCSF that contained the following (in mM): 125 NaCl, 25 NaHCO_3_, 2.5 KCl, 10 glucose, 2 CaCl_2_, and 2 MgCl_2_, pH 7.4, 300 mOsm. The bath temperature was turned off upon transfer to recording aCSF, and the solution was left to cool down to room temperature. Slices were allowed to recover for 30 min before experiments began, and acute slices were maintained at room temperature and continuously oxygenated for up to 7 h following the slicing procedure.

### Electrophysiology

Acute slices were transferred to a recording chamber, held under a harp, and continuously perfused with warm (32°C) and oxygenated recording aCSF. The CA1 region of the hippocampus was identified under an upright microscope (Scientifica) with a 4× objective [numerical aperture (NA) 0.1, Olympus] and neurons were identified under a 40× water-immersion objective (NA 0.8, Olympus) for visually guided whole-cell recordings. Recording electrodes were fabricated from borosilicate glass capillaries (BF150-86-10HP, Sutter Instrument) on a P-1000 micropipette puller (Sutter Instrument). Recording electrodes were filled with a solution containing the following (in mM): 130 Cs-methanesulfonate, 10 HEPES, 2 MgCl_2_.6H_2_O, 4 MgATP, 0.3 NaGTP, 7 disodium phosphocreatine, and 5 KCl, pH 7.3 and 290 mOsm. For data presented in Figure 7, the intracellular solution contained the following (in mM): 140 K-gluconate, 1.1 EGTA, 0.1 CaCl_2_, 10 HEPES, 2 MgATP, 0.3 NaGTP, pH 7.35 and 290 mOsm (Fraser and MacVicar, 1996). For experiments performed with intracellular BAPTA and presented in Figure S3, the solution contained the following (in mM): 90 Cs-methanesulfonate, 10 Cs_4_BAPTA, 10 HEPES, 2 MgCl_2_.6H_2_O, 4 MgATP, 0.3 NaGTP, 7 disodium phosphocreatine, and 5 KCl, pH 7.2 and 290 mOsm. Electrodes filled with these intracellular solutions had a resistance of 4–8 MΩ when inserted in the bath. The liquid junction potential was not corrected. Micromanipulators (Patchstar, Scientifica) were used to approach the recording electrodes to targeted neurons, and standard techniques were used to obtain whole-cell recordings. The electrophysiological signal was amplified (Multiclamp 700B, Molecular Devices) and digitized at 10 kHz (Digidata 1550B, Molecular Devices) before displaying and saving to a personal computer (Clampex v11.3, Molecular Devices). Only neurons for which the access resistance was <30 MΩ and with a resting membrane potential more hyperpolarized than −55 mV were included. Optogenetic stimulation was delivered from a pE-400 max CoolLED illumination system at 450 nm (CoolLED). Light power was measured under the objective with a PM101R power meter (Thorlabs). Salts used to prepare solutions for electrophysiological experiments were purchased from Sigma-Aldrich except for NaHCO_3_ (Thermo Fisher Scientific). The following compounds were also used in experiments: TTX (1 µM, tetrodotoxin with citrate, Biotium), apamin (100 nM, MedChemExpress), CdCl_2_ (100 µM, Thermo Fisher Scientific), and carbachol (CCh; 50 µM, Thermo Fisher Scientific).

### Two-photon Ca^2+^ imaging

Illumination was provided by a Chameleon Discovery NX femtosecond laser (80 MHz repetition rate, 100 fs pulse duration, Coherent) tuned at 810 nm. The laser was directed to a random-access two–photon microscope (AODScope, Karthala System). The laser position was controlled by a pair of acousto-optic deflectors in the *X*–*Y* planes and focused under a 25× water-immersion objective (NA 0.95, Leica). Transmitted and emitted photons were detected below and above the sample, each split in a green and a red channel and collected with four photomultiplier tubes (PMTs). For transmitted and emitted signal, photons were filtered through an IR blocking filter (TF1, Thorlabs) and split into two channels using a 562 nm dichroic mirror (Semrock). Green channel photons were filtered with a 510/84 nm bandpass filter and red channel photons were filtered with a 607/70 nm bandpass filter before being directed to two H12056P-40 PMTs (Hamamatsu) operating in photon counting mode. Transmitted and emitted signals were averaged in their respective channels before analyzing the data. Whole-cell recordings were obtained from CA1-PYRs using the Cs^+^-based intracellular solution detailed above and supplemented with Alexa Fluor 594 (40 µM) and Fluo-5F (385 µm). Recording sites were distributed across the dendritic arbor in a field of view. We typically recorded from 20 sites near-simultaneously with a 90 µs dwell time, yielding an average imaging speed of 500 Hz, similar to our previous studies employing random-access two–photon microscopy (Chamberland et al., 2014, 2017; Chamberland et al., 2018). Plateaus were terminated with mock inhibitory postsynaptic potentials (IPSPs). We observed that plateaus were generally harder to terminate in these conditions, possibly because of the added Ca^2+^ buffering capacity preventing *I*_SK_ activation. We used mock IPSPs with larger amplitude and longer offset to ensure terminations. This was also the case in the presence of TTX. Dendritic segments corresponded to 1–4 physical recording sites separated by <76 µm across a continuous dendritic branch.

### Single-cell labeling, confocal imaging, and anatomical tracing

Neurons filled with biocytin (1–3%, w/v) were fixed in 4% PFA dissolved in PBS. Biocytin was revealed in filled neurons using the procedure described before (Chamberland et al., 2023; 2024). Briefly, slices with biocytin-filled neurons were treated with H_2_O_2_ (30 min), Triton X-100 (1 h), and a streptavidin-conjugated Alexa Fluor 633 (1:100, incubated overnight). Slices were rinsed, mounted on microscope slides with coverslips, and allowed to rest for at least 2 weeks before confocal imaging. Confocal images were obtained on a Leica SP8 or Zeiss Axo Imager.Z2 upright confocal microscope with a 40× oil-immersion objective. Neurons were traced in Neurolucida 360 (v.2024.2.2, MBF Bioscience). The axonal distribution was measured in 10 µm bins starting from the pyramidal cell layer. Only neurons with total axonal length >450 µm were included for analysis presented in Figure 4*E*. For data presented in Figure 4, *A* and *B*, images were obtained on a Nikon A1R-HD confocal microscope with a 20× objective at 0.63 pixels/μm in *x*–*y* and 4 μm *z*-steps. Brains were serially sectioned in the coronal plane, and sections 100 μm apart were analyzed. Data from the left and right hemispheres were combined. Confocal scans encompassed the entirety of the 50 μm thickness of the CA1 hippocampus. Sample preparation, imaging, and cell quantification were performed similarly as described previously (Vaasjo et al., 2022).

### Data analysis and statistical treatment

Electrophysiological data were analyzed in Clampfit (v11.3, Molecular Devices), Igor Pro (v9.05, WaveMetrics), and Microsoft Excel (Microsoft). Ca^2+^ imaging data were exported to and analyzed in Microsoft Excel and Igor Pro. Images were analyzed in Fiji (ImageJ 1.54f). Normality of the data distribution was tested using the Shapiro–Wilk test. For normally distributed paired data, paired Student's *t* test was used. For non-normally distributed paired data, Wilcoxon signed-rank test was used. For normally distributed unpaired data, unpaired Student’s *t* test was used. For non-normally distributed unpaired data, a Mann–Whitney *U* test was used. Significance levels are reported everywhere as follows: n.s., nonsignificant; *p < 0.05; **p < 0.01; and ***p < 0.001. Statistical tests were performed in Python (Jupyter Notebook, v7.2.2) or in Clampfit.

### Biophysical modeling

A single-compartment conductance-based NEURON (v.8.2.6) model was assembled and controlled in Python (Hines and Carnevale, 1997; Hines et al., 2009). The compartment was a sphere with a diameter of 10 µm with surface area of 3.14 × 10^−6^ cm^2^. The membrane capacitance (*C*_m_) was set to 1 µF/cm^2^. The inhibitory synapse was connected in the middle of the compartment. The following mechanisms were included: passive leak (pas; Hines and Carnevale, 1997), voltage-gated calcium channel (cal, referred to as VGCC; Poirazi et al., 2003a,b), calcium-activated potassium current (kca; Destexhe et al., 1994), intracellular calcium dynamics (cad; Destexhe et al., 1993), and inhibitory synapse (ExpSyn, referred to as GABA; Hines and Carnevale, 1997). Simulations were performed at 32°C with a time step of 0.025 ms. The differential equation governing *V*_M_ is given as follows:

$$C_{m}\frac{dV}{dt}=\;-(I_{\mathrm{pas}}+I_{\mathrm{VGCC}}+I_{KCa}+I_{\mathrm{GABA}}-I_{\mathrm{inj}}),$$

with

$$I_{\mathrm{pas}}=\;g_{\mathrm{pas}}(V-\;E_{\mathrm{pas}}),$$

$$I_{\mathrm{VGCC}}=\;{\bar{g}}_{\mathrm{cal}}\cdot m\cdot h_{2}(ca_{i})\cdot GHK(V,ca_{i},ca_{o}),$$

$$I_{KCa}=\;{\bar{g}}_{kca}\cdot m^{3}\cdot (V-\;E_{K}),$$

$$I_{\mathrm{GABA}}=\;g_{\mathrm{GABA}}(V-\;E_{\mathrm{GABA}}).$$

For *I*_VGCC_, the differential equations for the gating variables *m*_cal_ are as follows:

$$\frac{dm}{dt}=\;\frac{m_{\infty }(V)-m}{\tau_{m}(V)},$$

with

$$m_{\infty }(V)=\;\frac{\alpha_{m}(V)}{\alpha_{m}(V)+\beta_{m}(V)},$$

$$\tau_{m}(V)=\;\frac{1}{tfa\;\cdot [\alpha_{m}(V)+\;\beta_{m}(V)]},$$

$$\alpha_{m}(V)=0.055\cdot \frac{-27.01-V}{\exp\;\left(\frac{-27.01-V}{3.8}\right)-1},$$

$$\beta_{m}(V)=\;0.94\cdot \exp\;\left(\frac{-63.01-V}{17}\right).$$

For $I_{KCa}$, the differential equation for the gating variable *m_kca_* is as follows:

$$\frac{dm}{dt}=\;\frac{m_{\infty }(ca_{i})-m}{\tau_{m}(ca_{i})},$$

with

$$m_{\infty }(ca_{i})=\;\frac{{\left(ca_{i}/ca_{c}\right)}^{2}}{1+{\left(ca_{i}/ca_{c}\right)}^{2}},$$

$$\tau_{m}(ca_{i})=\;\frac{1}{\beta \cdot (1+{\left(ca_{i}/ca_{c}\right)}^{2}\cdot t\mathrm{adj}}.$$

The [Ca^2+^]_i_ dynamics are governed by the differential equation as follows:

$$\frac{dca_{i}}{dt}=\;-\frac{10^{4}}{2F\cdot \mathrm{depth}}\cdot I_{\mathrm{VGCC}}+\frac{ca_{inf}-ca_{i}}{\tau_{r}},$$

where *ca_i_* is [Ca^2+^]_i_; *F* is the Faraday constant; depth is the depth of the submembrane shell in which Ca^2+^ accumulates (micrometer), here set to 0.1; *ca*_inf_ is steady-state [Ca^2+^]_i_; and *τ_r_* is Ca^2+^ removal time constant.

Conductances for VGCCs and SK were determined empirically. A grid search was performed to find combinations of parameters yielding plateaus with similar duration as observed experimentally (Fig. S4), and these parameters were generally comparable with previous reports (Migliore et al., 1999; Poirazi et al., 2003b; Combe et al., 2018). The biophysical model will be made available on modeldb at the time of publication.

## Results

### Dendritic inhibition terminates plateaus in CA1-PYRs

Plateau potentials are prolonged depolarizations in CA1-PYRs that control synaptic plasticity and memory formation (Golding et al., 2002; Takahashi and Magee, 2009; Bittner et al., 2017; Zhao et al., 2022). *Sst*-INs primarily synapse onto CA1-PYRs dendrites where they modulate local activity (Lacaille et al., 1987; Yanovsky et al., 1997; Lovett-Barron et al., 2012; Pelkey et al., 2017; Chamberland et al., 2024). How *Sst*-INs control ongoing plateaus in CA1-PYRs has yet to be elucidated.

**Figure 1.**
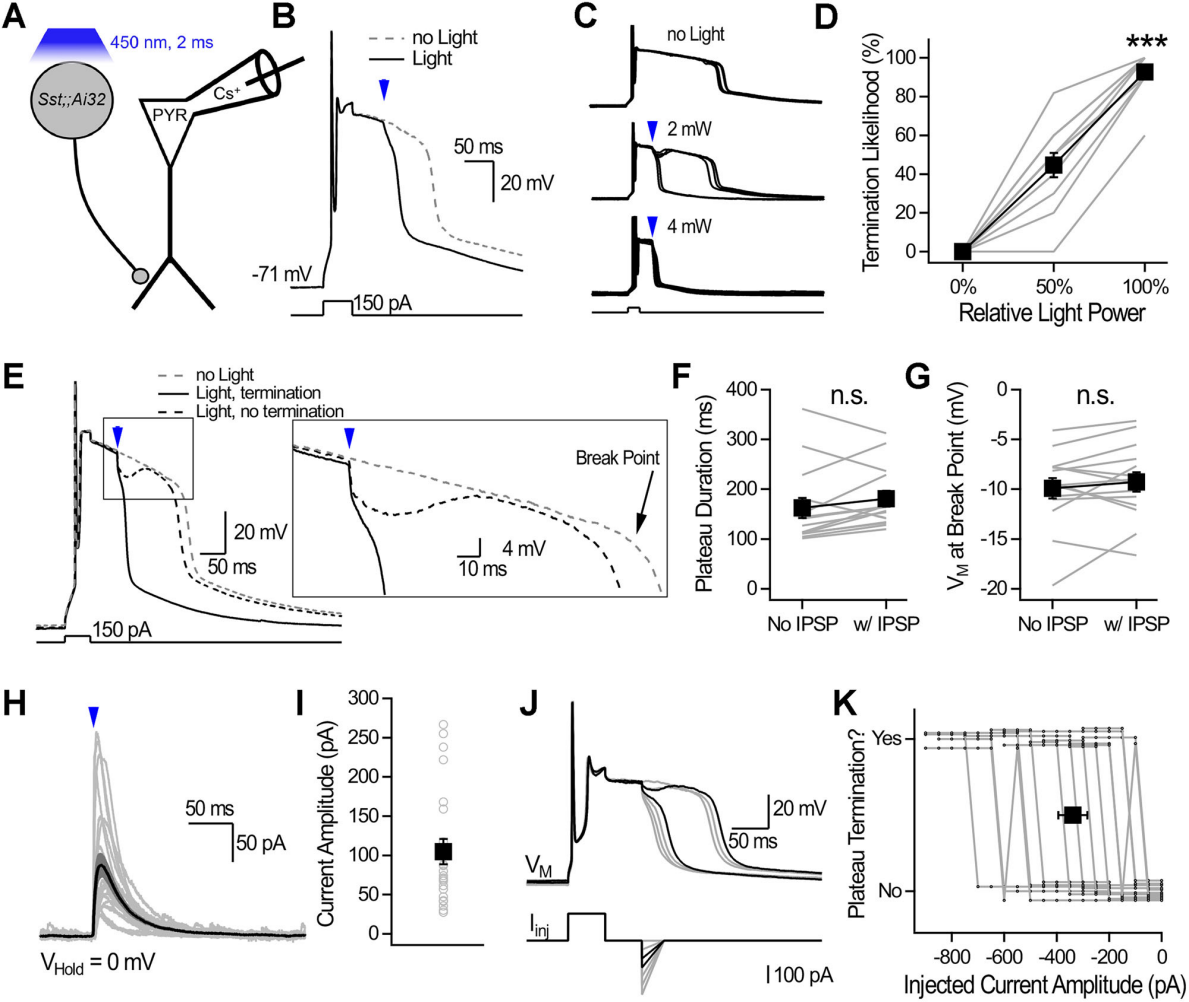
Optogenetic activation of *Sst*-INs terminates plateaus in an all-or-none manner in CA1-PYRs. ***A***, Scheme showing the recording configuration. ***B***, Current-clamp recording from a CA1-PYR. Brief current injections evoked long-lasting plateaus that were terminated by optogenetic stimulation of *Sst*-INs. ***C***, Current-clamp recordings from a CA1-PYR showing plateaus in the absence of light (top) and with the application of blue light (middle, 2 mW; bottom, 4 mW). Five consecutive trials are shown for each condition. ***D***, Termination likelihood as a function of light power. ***E***, Traces showing plateaus in the absence of light and with sub- and suprathreshold light. An IPSP is evident for subthreshold optogenetic stimulation. ***F***, Plateau duration in the absence of light (No IPSP) and with a subthreshold IPSP (w/ IPSP). ***G***, Membrane potential at natural break point for plateaus in the absence of light and with a subthreshold IPSP. ***H***, Voltage-clamp recordings (0 mV) showing IPSCs evoked by optogenetic stimulation with just-threshold light power for terminations. Gray traces show individual neuron average, and the black trace shows the population average ± SEM (*n* = 22 neurons). ***I***, Distribution of IPSC amplitudes for data shown in ***H***. Gray circles show individual neurons, and the black square shows average ± SEM (*n* = 22). ***J***, Current injection mimicking IPSPs terminate plateaus. Example shows seven consecutive traces for which the mock IPSP amplitude was gradually increased. Black traces show the just sub- and suprathreshold conditions. ***K***, Stepwise increases in mock IPSP amplitude elicited an all-or-none termination of plateau potentials in individual neurons. Light gray traces show individual neurons (*n* = 14), and the black square indicates the average ± SEM termination threshold, determined from sigmoid fits to individual neurons. For ***D, F, G***, gray lines show individual neurons, and the black trace shows average ± SEM. n.s., nonsignificant; ***p < 0.001.

We performed whole-cell recordings from deep CA1-PYRs with Cs^+^-based intracellular solution in acute hippocampal slices from *Sst-Cre;;Ai32* mice which express channelrhodopsin-2(H134R) in *Sst*-INs (Fig. 1*A*; Chamberland et al., 2023). Current injection (50 ms; 50–200 pA; 118.2 ± 4.6 pA; *n* = 51) generated a single or few APs followed by plateaus of long duration (140.2 ± 10.2 ms; *n* = 51; Fig. 1*B*). Optogenetic activation of *Sst*-INs with a brief pulse of blue light (2 ms, 450 nm) terminated plateaus in an all-or-none manner (Fig. 1*B*). Light power was adjusted at the threshold value to observe terminations (0.7 ± 0.2 mW; 44.7 ± 6.3%; *n* = 11) and doubling the light power significantly increased the likelihood of plateau termination (1.4 ± 0.4 mW; 92.7 ± 3.4%; *n* = 11; *p* < 0.001; paired Student's *t* test; Fig. 1*C*,*D*). In cases where optogenetic stimulation failed to terminate plateaus, an IPSP was visible and the *V*_M_ returned to the depolarized phase where the plateau pursued its normal course as if the IPSP-induced disturbance had no lasting impact (plateau duration without IPSP = 162.4 ± 20.1 ms vs with IPSP = 180.9 ± 15.9 ms; *n* = 14; *p* = 0.15, Wilcoxon signed-rank test; Fig. 1*E*,*F*; break point without IPSP = −9.9 ± 1 mV vs with IPSP = −9.3 ± 1 mV; *n* = 14; *p* = 0.33, paired Student's *t* test; Fig. 1*G*). Inspection of individual traces revealed that terminations happened around a similar point in *V*_M_ suggesting that a putative threshold had to be reached (Fig. 1*C*,*E*). IPSCs evoked by optogenetic stimulation at the light intensity corresponding to the plateau termination threshold and measured in voltage clamp at 0 mV were of moderate amplitude and decayed with a time constant of 28.1 ± 1.6 ms (*n* = 22 neurons; Fig. 1*H*,*I*). These results show that *Sst*-INs terminate plateaus in an all-or-none manner in CA1-PYRs.

Optogenetic stimulation caused IPSPs that invariably preceded plateau termination (Fig. 1*B*,*C*,*E*). We tested whether membrane hyperpolarization suffices to terminate plateaus by injecting IPSP-mimicking current waveforms via the recording electrode. Mock IPSPs were approximated by an instantaneous hyperpolarizing current of increasing amplitude that decayed over 30 ms (Fig. 1*J*). Mock IPSPs terminated plateaus in an all-or-none manner (Fig. 1*J*,*K*). This threshold-like behavior was evident in all neurons tested (*n* = 14/14), where stepwise increases in injected current produced an abrupt transition from plateau persistence to termination. Overall, these results show that while inhibition provides the necessary membrane hyperpolarization, plateau termination is operated by a cell-autonomous mechanism.

### Plateaus become progressively more susceptible to termination

The duration of plateaus recorded in CA1-PYRs is variable (Epsztein et al., 2011; Bittner et al., 2015). Excitatory synapses formed by CA1-PYRs on *Sst*-INs demonstrate modest quantal size but show considerable short-term facilitation, suggesting that *Sst*-INs could be optimally recruited during CA1-PYRs repetitive firing (Ali and Thomson, 1998; Losonczy et al., 2002; Pouille and Scanziani, 2004; Biro et al., 2005; Chamberland et al., 2024). Consequently, dendritic inhibition mediated by *Sst*-INs could occur at multiple time points during plateaus.

**Figure 2.**
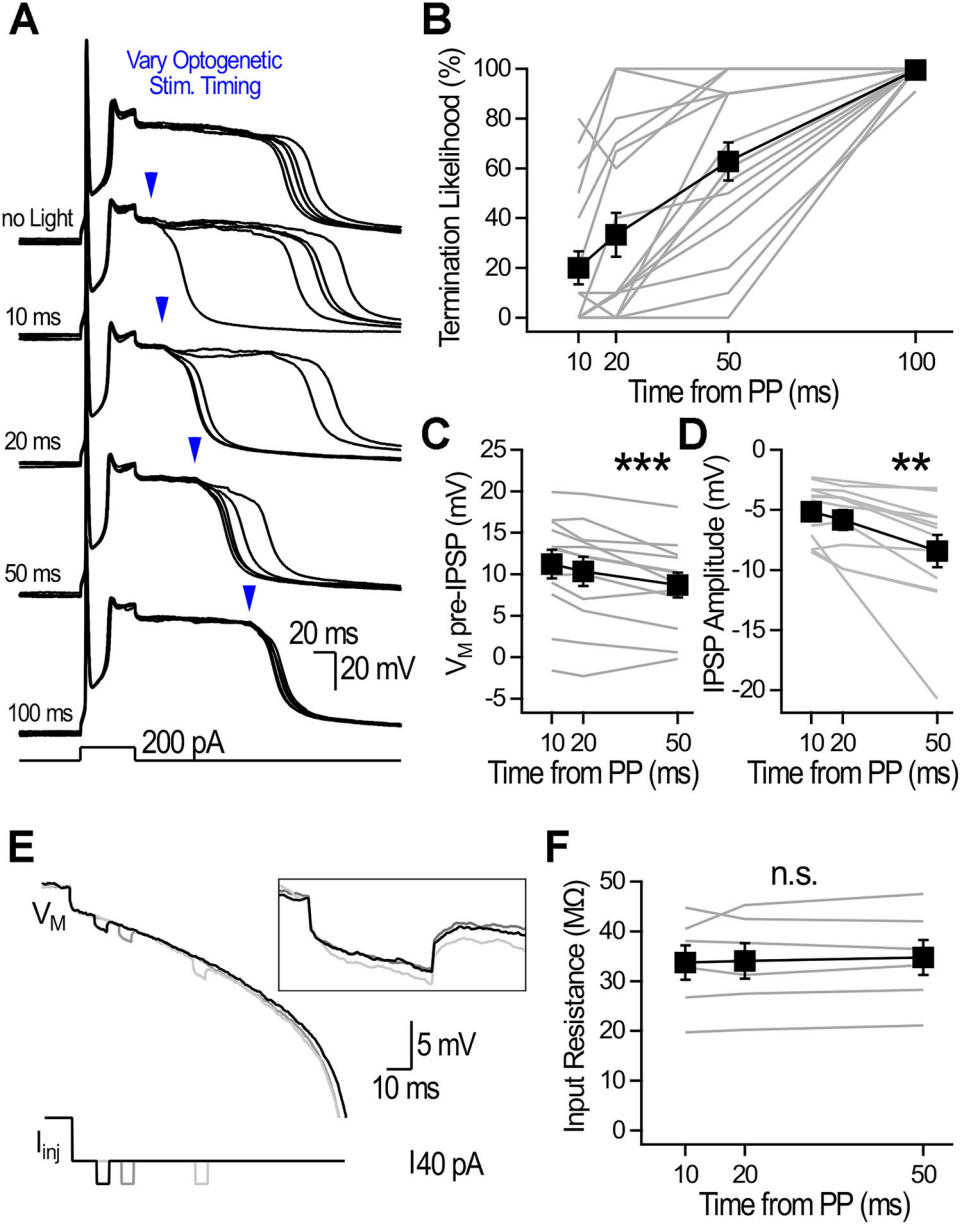
Plateaus become gradually more sensitive to inhibition. ***A***, Plateaus without light application (top) and with optogenetic stimulation delivered at four timepoints during plateaus. Examples shown are five consecutive traces for each condition. Light power was adjusted at 50 ms to obtain both success and failures and was kept constant for all measurements within a neuron. ***B***, Termination likelihood of plateau potentials (PP) as a function of optogenetic timing. ***C***, Membrane potential just before the IPSP onset for 10, 20, and 50 ms timepoints. ***D***, IPSP amplitude for subthreshold IPSPs evoked at 10, 20, and 50 ms timepoints. ***E***, Brief current injection via the recording pipette at 10, 20, and 50 ms following plateau induction. There was no difference in the input resistance evolution whether the injected current was depolarizing or hyperpolarizing, and results were pooled. ***F***, The measured input resistance did not change as a function of current injection timing. **p < 0.01; ***p < 0.001.

Optogenetic stimulation of *Sst*-INs was delivered 50 ms following plateau induction, and the light power was adjusted near the termination threshold in each cell (light power, 3.9 ± 1.3 mW; termination likelihood, 62.7 ± 7.7%; *n* = 17; Fig. 2*A*,*B*). Identical optogenetic stimulation delivered at 10, 20, 50, and 100 ms during the plateau revealed a significant effect of stimulation timing on termination likelihood (Friedman test, *χ*^2^ = 42.8; *p* < 10^−8^), with earlier illumination being significantly less effective at terminating plateaus (10 ms, 20 ± 6.7%; *p* < 0.01; 20 ms, 33.3 ± 8.8%; *p* < 0.01; both compared with 50 ms, 62.7 ± 7.7%; *n* = 17; post hoc Wilcoxon signed-rank tests with Holm–Bonferroni correction; Fig. 2*B*). These results show that plateaus become increasingly susceptible to termination as they progress in time.

We next investigated why early resistance to inhibition gradually dissipates. We analyzed trials in which plateaus were not terminated and measured: (1) the *V*_M_ right before optogenetic stimulation and (2) the light-evoked IPSP amplitude as a function of time from plateau onset. A significant hyperpolarizing shift in *V*_M_ during plateaus (*n* = 12; Fig. 2*C*) was paralleled by gradually larger IPSP amplitudes (*n* = 12; Fig. 2*D*). The observed increase in IPSP amplitude is inconsistent with the decrease in Cl^−^ driving force caused by the gradual hyperpolarizing shift in *V*_M_, prompting us to examine the evolution of membrane resistance. Brief current steps delivered via the recording pipette at corresponding timepoints revealed that the input resistance remained constant during plateaus (repeated-measures ANOVA, *F*_(2,10)_ = 0.017; *p* = 0.98; Fig. 2*E*,*F*). Overall, these results indicate that feedback inhibition governs plateau duration, with progressive *V*_M_ hyperpolarization and increasing IPSP amplitude both enhancing susceptibility to termination over time.

### OLM^Ndnf^ preferentially terminate plateaus compared with OLM^α2^

*Sst*-INs are genetically, anatomically, and functionally diverse (Somogyi and Klausberger, 2005; Katona et al., 2014; Booker and Vida, 2018; Chamberland et al., 2024). A proportion of *Sst*-INs located in stratum oriens project their axon to stratum lacunosum-moleculare (OLMs; Lacaille et al., 1987; Naus et al., 1988; Schwartzkroin et al., 1990; Baude et al., 1993; McBain et al., 1994). OLM^Ndnf^ are a subpopulation of OLMs which preferentially innervate CA1-PYRs over fast-spiking INs, unlike OLM^α2^, which innervate both targets without such selectivity (Leao et al., 2012; Chamberland et al., 2024).

**Figure 3.**
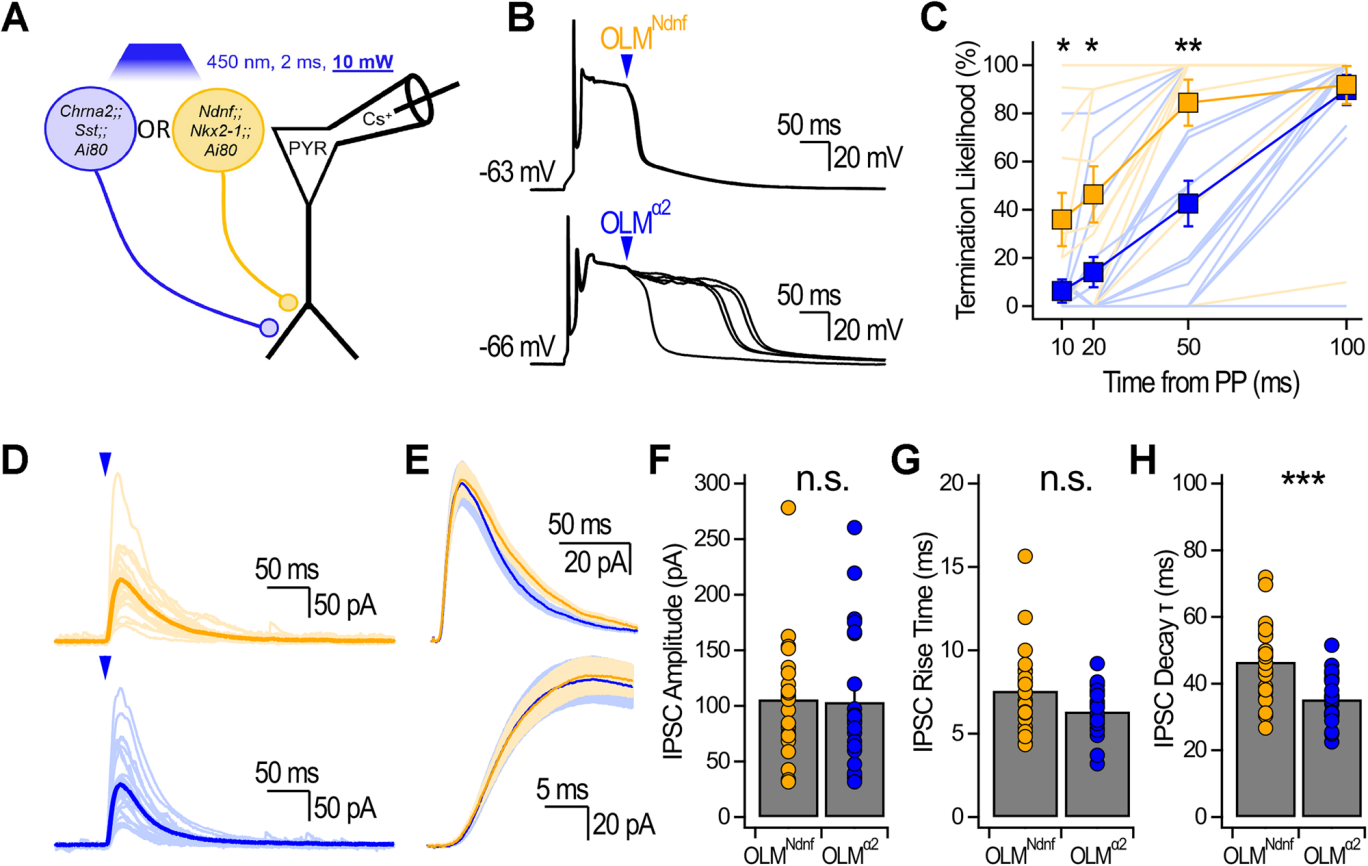
OLM^Ndnf^ terminate plateaus more effectively than OLM^α2^. ***A***, Scheme showing the recording configuration. The light power was kept constant at 10 mW across all CA1-PYRs recorded in both transgenic mouse models. ***B***, Example traces showing the impact of optogenetic stimulation of OLM^Ndnf^ and OLM^α2^. Five consecutive traces are shown. ***C***, Termination likelihood as a function of optogenetic stimulation timing. For the 10 ms timepoint, OLM^Ndnf^, 35.9 ± 11.1%; *n* = 11; OLM^α2^, 6.3 ± 4.8%; *n* = 16, *p* < 0.05, Mann–Whitney *U* test. For the 20 ms timepoint, OLM^Ndnf^, 46.4 ± 11.7%; *n* = 11; OLM^α2^, 14.2 ± 6.3%; *n* = 16, *p* < 0.05, Mann–Whitney *U* test. ***D***, IPSCs recorded in voltage clamp (0 mV) evoked by constant optogenetic stimulation. Light color traces show the average for individual neurons. Population averages are shown in darker traces with shaded area showing ±SEM. ***E***, Population averages at two timescales to show differences in decay and in rise time. Shaded areas show ±SEM. ***F***, Optogenetically evoked IPSCs amplitude recorded for OLM^Ndnf^ and OLM^α2^. ***G***, IPSC rise time (20–80% of maximum amplitude) for both OLM subtypes. ***H***, IPSC decay *τ* for both OLM subtypes. n.s. = nonsignificant; *p < 0.05; **p < 0.01; ***p < 0.001.

To investigate how OLM subtypes control plateaus, we generated *Ndnf-FlpO;;Nkx2-1-Cre;;Ai80* and *Chrna2-Cre;;Sst-FlpO;;Ai80* mice. Optogenetic stimulation was kept constant across all experiments (10 mW; 2 ms) and delivered 10, 20, 50, and 100 ms after plateau initiation (Fig. 3*A*–*C*). As expected, termination likelihood for both OLM^Ndnf^ and OLM^α2^ increased as a function of time during plateaus (Fig. 3*C*). Interestingly, termination likelihood was higher for OLM^Ndnf^ than OLM^α2^, an effect that was significant at the 10, 20, and 50 ms timepoints (for the 50 ms timepoint, OLM^Ndnf^, 84.4 ± 9.6%, *n* = 11; OLM^α2^, 42.6 ± 9.4%; *n* = 16; *p* < 0.01; Mann–Whitney *U* test; Fig. 3*C*). To investigate why OLM^Ndnf^ were more likely to terminate plateaus, we recorded inhibitory postsynaptic currents (IPSCs) in CA1-PYRs voltage-clamped at 0 mV (Fig. 3*D*,*E*). Optogenetic stimulation of OLM^Ndnf^ and OLM^α2^ evoked IPSCs of similar amplitude and rise time (amplitude, OLM^Ndnf^, 105.9 ± 10.7 pA; *n* = 23; OLM^α2^, 103.4 ± 13.3 pA; *n* = 22; *p* = 0.6; Mann–Whitney *U* test; Fig. 3*F*; rise time, OLM^Ndnf^, 7.6 ± 0.5 ms; *n* = 23; OLM^α2^, 6.4 ± 0.3 ms; *n* = 22; *p* = 0.1; Mann–Whitney *U* test; Fig. 3*G*). However, the decay kinetics of IPSCs evoked by OLM^Ndnf^ photostimulation were significantly slower than those evoked by identical photostimulation of OLM^α2^ (decay *τ*, OLM^Ndnf^, 46.5 ± 2.4 ms; *n* = 23; OLM^α2^, 35.4 ± 1.5 ms; *n* = 22; *p* < 0.001; unpaired Student's *t* test; Fig. 3*H*). Overall, these results show that OLM^Ndnf^ and OLM^α2^ can terminate plateaus, but OLM^Ndnf^ do so more effectively.

**Figure 4.**
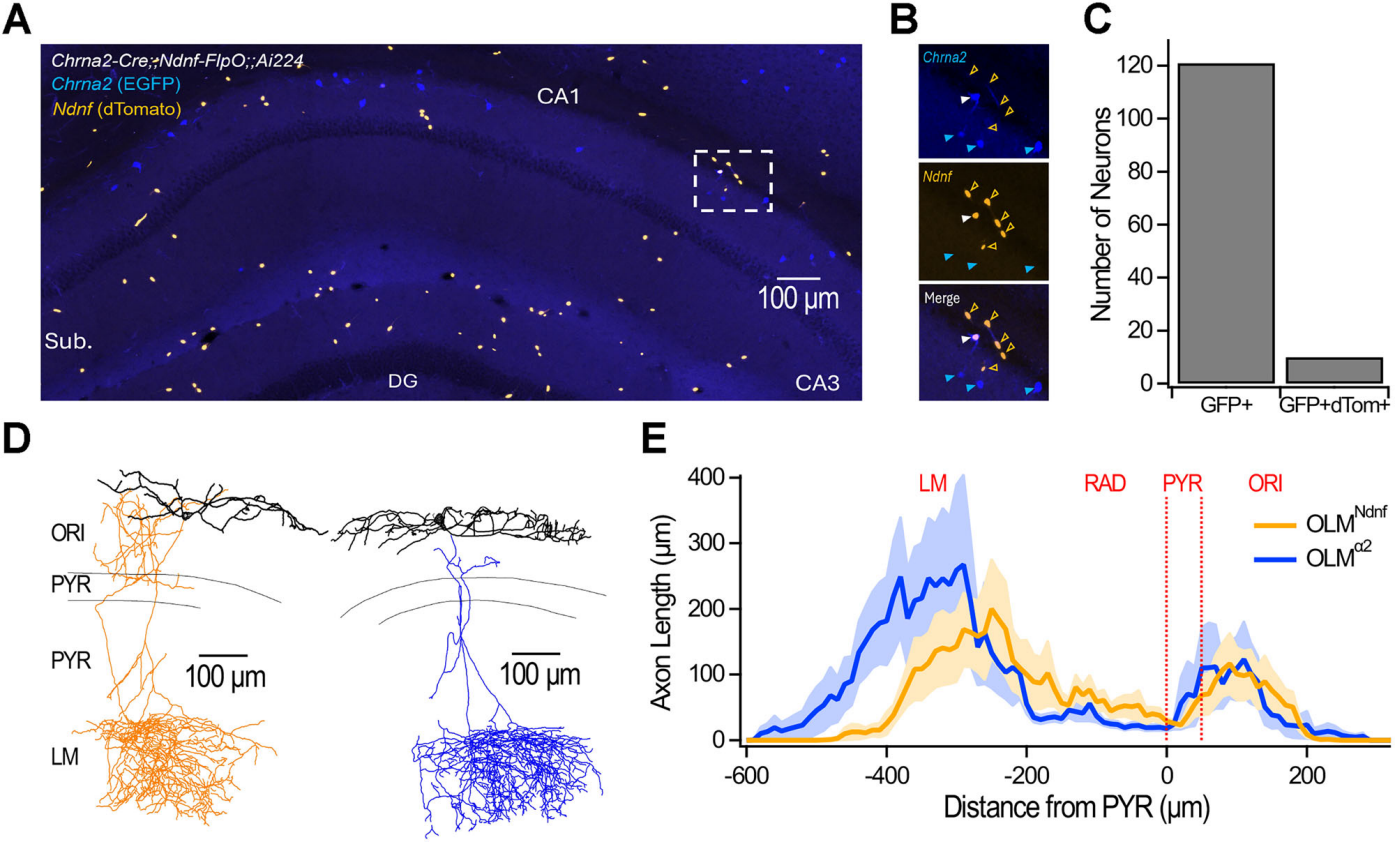
OLM^Ndnf^ and OLM^α2^ minimally overlap and possess distinct axonal arborizations. ***A***, Confocal image obtained from a hippocampal section (50 µm) of a triple transgenic *Chrna2-Cre;;Ndnf-FlpO;;Ai224* mouse. ***B***, Insets show examples of overlapping and nonoverlapping neurons. Note that while dTomato is restricted to the soma, GFP expression is also seen in the cytosol as reported before for this reporter line (Ben-Simon et al., 2024). ***C***, Quantification of overlapping and nonoverlapping neurons sampled across multiple images. Because *Ndnf*-expressing neurons in stratum oriens and in the alveus could be from a different subtype (such as neurogliaform), we chose to report the number of GFP cells that coexpress dTomato. ***D***, Neurolucida reconstructions of an OLM^Ndnf^ and an OLM^α2^ recorded in *Ndnf-FlpO;;Nkx2-1-Cre;;Ai65* and *Chrna2-Cre;;Ai9* transgenic animals, respectively. ***E***, Axonal length as a function of the distance from the pyramidal cell layer. Dark lines show the average across all cells (*n* = 13 for OLM^Ndnf^ and *n* = 10 for OLM^α2^), and shaded areas show SEM. All neurons reconstructed and reported here are shown in Figures S1 and S2.

Functional differences prompted us to more closely evaluate the identity of OLM^Ndnf^ and OLM^α2^ which show transcriptomic proximity and adopt a common anatomical phenotype (Harris et al., 2018; Chamberland et al., 2024). To test how this translates in situ with the transgenic mouse models, we generated triple transgenic *Ndnf-FlpO;;Chrna2-Cre;;Ai224* mice to drive the nuclear expression of EGFP and dTomato as a function of Cre and FlpO expression, respectively (Ben-Simon et al., 2024; Fig. 4*A,B*). Analysis of confocal images revealed that EGFP and dTomato expression was distributed as expected from previous data in *Chrna2*-Cre mice (Leao et al., 2012) and *Ndnf* transcripts in the hippocampus (Allen Institute for Brain Science, 2025; Fig. 4*A,B*). We found that only 8.3% of GFP-expressing cells in stratum oriens coexpressed dTomato (Fig. 4*C*). We chose this quantification approach because *Ndnf* is expressed in multiple IN subtypes outside of the *Nkx2-1* intersection that targets OLM^Ndnf^, while *Chrna2* on its own targets OLM^α2^ (Leao et al., 2012; Chamberland et al., 2024). Minimal colocalization between the two fluorescent proteins suggested that OLM^Ndnf^ and OLM^α2^ are nearly nonoverlapping subpopulations. We next evaluated potential differences in axonal projections that could help account for the distinct IPSCs evoked by OLM^Ndnf^ and OLM^α2^ with biocytin fills of genetically targeted neurons followed by Neurolucida reconstructions (Fig. 4*D*; Figs. S1, S2). Axonal distribution analysis through the CA1 layers revealed that OLM^α2^ axons penetrated deeper in LM than those of OLM^Ndnf^ (Fig. 4*E*). Overall, OLM^Ndnf^ and OLM^α2^ are distinct subpopulations of OLMs that terminate plateaus, but OLM^Ndnf^ do so more effectively. OLM^Ndnf^ mediate slower IPSCs, a finding that is unlikely to be explained by differences in electrotonic attenuation along CA1-PYR dendrites.

### Plateaus require VGCCs but not voltage-gated Na^+^ channels

We sought to dissect the biophysical mechanisms underlying plateau termination through experiments and modeling. The constant input resistance observed during plateaus argues that plateaus are unlikely to originate from a single conductance that gradually decays over time but instead reflects a near-equilibrium between multiple conductances (Fig. 2*E*). Dendritic plateaus are abolished by application of VGCCs blockers and prolonged by SK channel blockade (Fraser and MacVicar, 1996; Wei et al., 2001; Cai et al., 2004). In addition, TTX-sensitive Na^+^ channels have been shown to contribute to multiple forms of regenerative activity in CA1-PYR dendrites (Magee and Johnston, 1995; Golding and Spruston, 1998; Cai et al., 2004; Takahashi and Magee, 2009). We next pharmacologically dissected the roles of these currents in plateaus and their termination.

**Figure 5.**
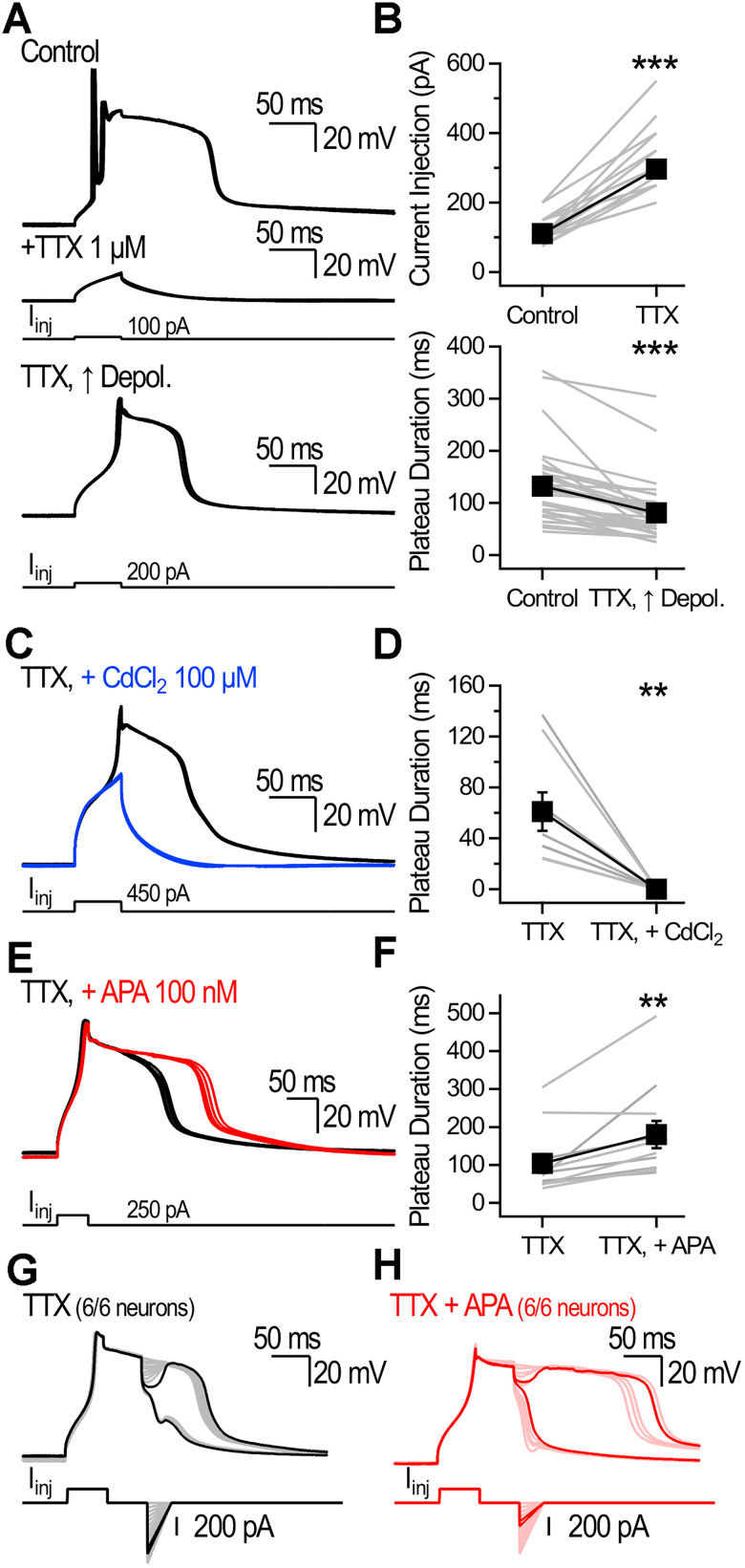
Pharmacological dissection of plateaus. ***A***, Plateaus recorded in control (top) and in the presence of TTX (1 µM). APs and plateaus were initially abolished (middle). Increasing the current injection amplitude recovered the plateaus but not the APs (bottom). Examples show five consecutive traces. ***B***, Just-suprathreshold current injection for neurons recorded in control and following bath application of TTX (top). Plateau duration in control and following recovery by increased depolarization in TTX (bottom). ***C***, Plateaus recorded in the presence of TTX (black) and following the addition of CdCl_2_ (100 µM, blue). Five consecutive traces are shown for both. ***D***, Plateau duration recorded in the presence of TTX and in the presence of TTX + CdCl_2_. ***E***, Plateaus recorded in the presence of TTX (black) and following perfusion of TTX + apamin (APA, 100 nM, red). ***F***, Plateau duration measured in TTX and following the application of TTX + APA. ***G***, ***H***, Plateaus were terminated by mock IPSPs in the presence of TTX (6/6 neurons) and in the presence of TTX + apamin (6/6 neurons). Examples show successive sweeps in which the mock IPSP amplitude was gradually increased. Dark lines show the just-subthreshold and just-suprathreshold trials for the *V*_M_ recordings and the injected current.

Bath application of the Na^+^ channel blocker TTX (1 µM) blocked APs and plateau generation in all neurons tested (*n* = 32). Increasing the current injection amplitude (control, 110.9 ± 5.2 pA; TTX, 296.1 ± 12.5 pA; *n* = 32; *p* < 0.001, Wilcoxon signed-rank test; Fig. 5*A*,*B*) consistently rescued plateaus without restoring APs (plateau likelihood, control, 99.5 ± 0.4%; TTX, 0%; increased current injection in TTX, 98.7 ± 0.8%; *n* = 31; Fig. 5*A*,*B*). Plateaus evoked in TTX were significantly briefer than in control condition (control, 132.4 ± 13 ms; TTX, 81.3 ± 10.2 ms; *n* = 32; *p* < 0.001, Wilcoxon signed-rank test; Fig. 5*B*), indicating that while TTX-sensitive Na^+^ currents are not essential for plateau generation, they likely enhance local depolarization.

Application of Cd^2+^ (100 µM) following TTX abolished plateaus (TTX, 61 ± 15 ms; TTX + CdCl_2_, 0 ms; *n* = 8; *p* < 0.01, Wilcoxon signed-rank test; Fig. 5*C*,*D*). Increasing the injected current (333.3 ± 28.1 pA) by more than threefold (1058.3 ± 96.8 pA) failed to rescue plateaus (*n* = 6), consistent with the necessity of VGCCs. Application of the SK channel blocker apamin (APA, 100 nM) after TTX significantly extended plateau duration (TTX, 104.1 ± 24.9 ms; TTX + APA, 179.7 ± 36.2 ms; *n* = 11; *p* < 0.01, Wilcoxon signed-rank test; Fig. 5*E*,*F*). Because intracellular Cs^+^ could impact SK function, we performed experiments to validate the presence of SK-mediated currents under our recording conditions. In whole-cell voltage–clamp recordings, a depolarizing step from −70 mV to 0 mV in the presence of TTX (1 µM) evoked a net outward current that was significantly reduced by apamin (*n* = 5; *p* < 0.01; paired Student's *t* test; Fig. S3*A*,*C*). The apamin-sensitive current rose gradually during the step depolarization (Fig. S3*D*). When recordings were performed with the calcium chelator BAPTA (10 mM) in the pipette, the net outward current was sharply reduced (*n* = 5; *p* < 0.01; unpaired Student's *t* test; Fig. S3*B*,*C*). Moreover, the apamin-sensitive current was fully prevented when CA1-PYRs were recorded with the BAPTA-containing intracellular solution (*n* = 5; *p* = 0.4; paired Student's *t* test; Fig. S3*C*,*D*). These results show a gradually rising, apamin-sensitive, Ca^2+^-dependent outward current consistent with SK channel function.

Importantly, mock IPSPs terminated plateaus in the presence of TTX (*n* = 6/6 cells tested; Fig. 5*G*) and in TTX + APA (*n* = 6/6 cells tested; Fig. 5*H*). These results show that although TTX-sensitive and apamin-sensitive currents modulate plateau dynamics, they are not required for IPSP-driven plateau termination. Overall, these results point to the central role of VGCCs in plateaus and their termination.

### Biophysical modeling provides a mechanistic explanation for plateau termination

**Figure 6.**
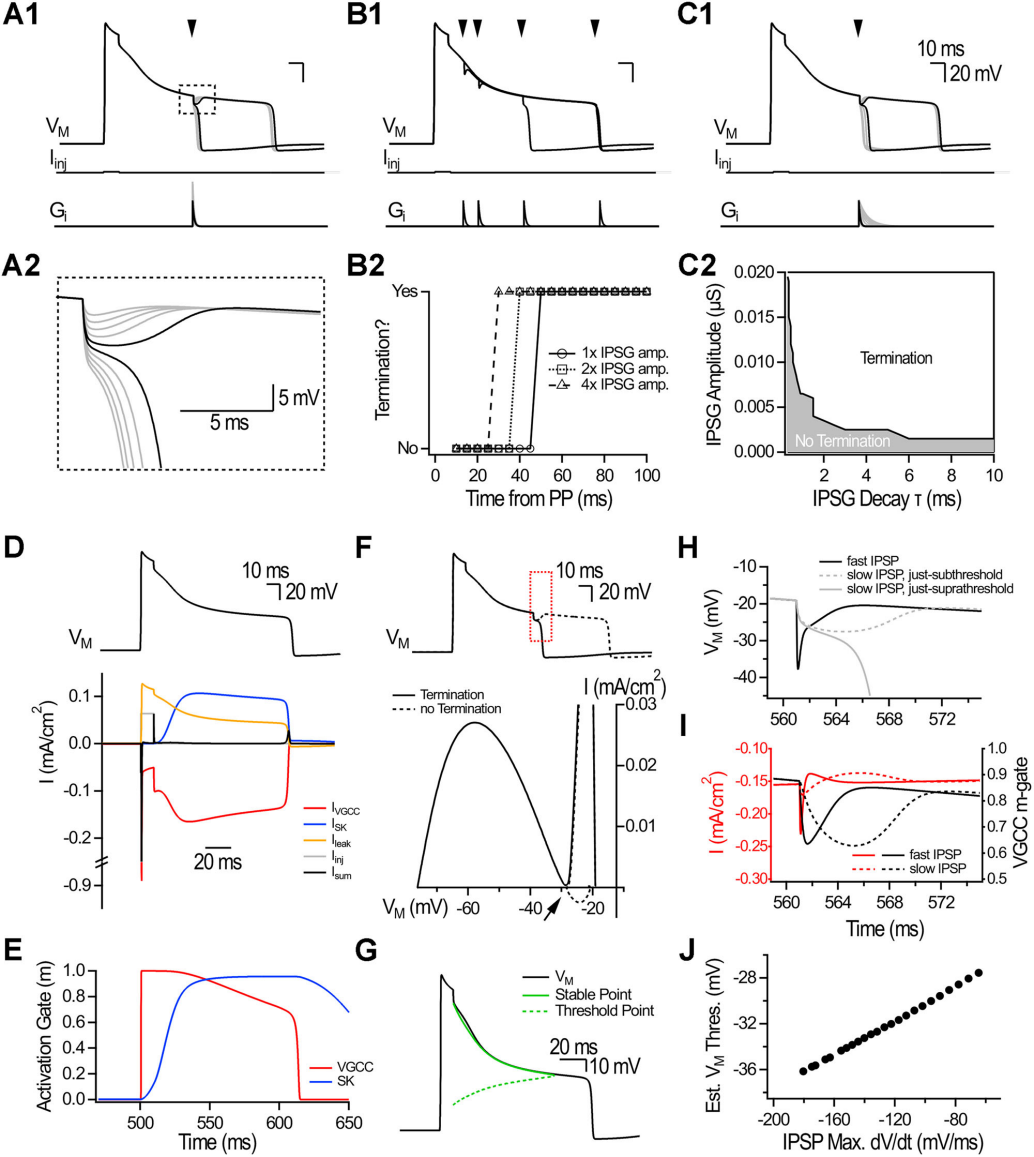
Single-compartment conductance-based model reveals the biophysical mechanisms of plateau termination. ***A1***, Simulations showing plateaus generated by brief current injection. An IPSG was delivered 50 ms following plateau initiation. Gradually increasing the IPSG amplitude revealed all-or-none termination. ***A2***, The blown-up boxed region from A1 shows the transition from nonterminating to terminating IPSPs. ***B1***, Constant IPSG delivered at 10, 20, 50, and 100 ms (indicated by arrowheads) during the plateau. The IPSG amplitude was initially adjusted to the just-threshold value for plateau termination at 50 ms. ***B2***, Termination as a function of IPSG timing (5 ms increment) for the data presented in B1, and for IPSGs with 2× (squares) and 4× (triangles) amplitude. ***C1***, Plateaus were challenged with IPSG of increasing decay *τ*. ***C2***, Results from simulations in which the parameter space for IPSG amplitude and decay *τ* was explored. ***D***, Dissection of currents (bottom) during simulated plateau (top). Currents are color-coded according to the legend in the figure. ***E***, Activation gate (m) for the VGCC (red) and SK (blue) conductances. ***F***, Just-subthreshold (dotted line) and just-suprathreshold (full line) IPSPs evoked by an IPSG delivered at 50 ms during the plateau. *I*–*V* curves for the two traces shown above, focusing on the region identified by the red box starting at the IPSP initiation. The arrow points to the *I* = 0 line crossing, a repelling point in *V*_M_. ***G***, Solving the HH equation for *I* = 0 reveals two sets of *V*_M_ values within the plateau physiological range. Inferred stable point (green full line) and inferred threshold point (green dotted line). ***H***, Simulations specifically selected to show how a fast IPSP (black line) can reach *V*_M_ values much more hyperpolarized without terminating the plateau than a slower IPSP. For the slow IPSP, the just-subthreshold (dotted gray line) and just-suprathreshold (full gray line) conditions are shown. ***I***, VGCC current and m-gate evolution for the nonterminating examples presented above. ***J***, Estimated termination threshold as a function of the IPSP maximum dV/dt. This estimation is obtained from pairs of just-subthreshold and just-suprathreshold values, where the estimated termination threshold corresponds to the most hyperpolarized *V*_M_ value reached during the just-subthreshold IPSP, and the IPSP maximum dV/dt is measured from the just-suprathreshold IPSP.

We next aimed to understand the biophysical mechanisms controlling plateau termination. The combination of VGCCs and SK currents (*I*_VGCC_ and *I*_SK_, respectively) with opposite polarity could support a depolarized state. A single-compartment model with VGCCs and SK conductances generated plateaus that outlasted the current injection before spontaneously repolarizing to resting *V*_M_ (Fig. 6*A*; Fig. S4). The model captured key experimental findings: (1) IPSPs terminated plateaus in an all-or-none manner (Figs. 1, 6*A*1,*A*2); (2) plateaus were initially resistant to inhibition but became increasingly susceptible to termination over time (Figs. 2, 6*B*1,*B*2); and (3) slow IPSPs terminated plateaus more effectively (Figs. 3, 6*C*1,*C*2).

First, an inhibitory conductance (IPSG) was injected at 50 ms during the plateau (Fig. 6*A*1). Gradually increasing IPSG amplitude revealed all-or-none termination. Failures to terminate plateaus were characterized by an IPSP waveform and plateaus continued their course seemingly unaffected (Fig. 6*A*1,*A*2). Second, the IPSG amplitude was adjusted at the just-threshold value to terminate plateaus at 50 ms and then injected at multiple timepoints in 5 ms increments from plateau generation (Fig. 6*B*1,*B*2). Plateaus were initially resistant to inhibition but became increasingly susceptible to termination as they progressed. IPSGs with larger amplitudes terminated plateaus earlier (Fig. 6*B*2). Third, we systematically swept through pairs of IPSG amplitude and decay time constant (*τ*) to identify multiple parameter pairs sufficient to terminate plateaus (Fig. 6*C*1,*C*2). For a given IPSG amplitude, slower IPSGs were more likely to terminate plateaus (Fig. 6*C*2). Therefore, a simple model incorporating VGCC and SK conductances recapitulated the main experimental findings.

We next used the model to understand plateaus in the absence of inhibitory events. Dissection of current dynamics revealed that during the plateau, *I*_VGCC_ was pitted against *I*_SK_ and *I*_leak_, such that the outward and inward currents nearly canceled each other (Fig. 6*D*). *I*_VGCC_ sustained *V*_M_ depolarization, which in turn kept VGCC activated, forming a positive feedback loop. As [Ca^2+^]_i_ rose, *I*_SK_ gradually increased (aligned with our experimental demonstration; Fig. S3), *V*_M_ slowly hyperpolarized, and *I*_sum_ trended closer to the *I* = 0 line without crossing it (Fig. S5*A*–*G*). This interaction suggested a near-stable point in *V*_M_. Plateaus ended spontaneously when *V*_M_ exited the range of value allowing sustenance of the positive feedback loop. The end of the plateau was characterized by a sharp drop in the VGCC m-gate and a large outward current unmasked by the abrupt loss of *I*_VGCC_ (Fig. 6*E*). These results indicate that plateaus are a near-stable point in *V*_M_ and that VGCC deactivation marks the end of the plateau.

We analyzed inhibition-driven terminations by comparing supra- and subthreshold IPSPs (Fig. 6*F*). In simulations with suprathreshold IPSPs, the *I*–*V* trajectories approached but did not cross the *I* = 0 line, eventually producing a net outward current that brought *V*_M_ back to its resting value. Like spontaneously ending plateaus, this was characterized by a sharp drop in the VGCC m-gate and unmasking of a large outward current (Fig. 6*F*; Fig. S6*A–C*). In contrast, subthreshold IPSPs showed *I*–*V* trajectories that crossed the *I* = 0 line, reversed slope, and returned *V*_M_ to the plateau state. This suggested the existence of a repelling (threshold) point in *V*_M_ that underlies all-or-none terminations (Fig. 6*F*). The reversal described above occurred because the increased Ca^2+^ driving force and reduced K^+^ driving force rebalanced the currents, opposing the IPSC and restoring the plateau (Fig. S6*A*–*F*). When the IPSC did not sufficiently disrupt this balance, m could catch up to its steady-state value (*m*_inf_), sustaining the plateau (Fig. S6*F*). Thus, for IPSCs to terminate plateaus, they must overcome this dynamic rebalancing to break the positive feedback loop maintaining VGCC activation. These findings provide a mechanistic explanation for all-or-none plateau termination.

Coexistence of stable and threshold points describes plateau behavior in cardiac Purkinje fibers (Noble and Tsien, 1969). To examine their evolution during plateaus in CA1-PYRs, we solved the Hodgkin–Huxley equation for *I* = 0 by assuming steady-state conditions (Fig. 6*G*). We identified two sets of gradually evolving *V*_M_ values within the plateau range that balanced the total membrane current (Fig. 6*G*). The first set of values (solid green line) was tracked by *V*_M_, consistent with the idea that plateaus are a gradually hyperpolarizing near-stable point in *V*_M_. The second set (dotted green line) initially resided at more hyperpolarized levels but gradually drifted toward more depolarized levels. This is the approximative threshold point, and its evolution explains why plateaus display an early resistance to inhibition that gradually fades.

Why are slow IPSPs better at terminating plateaus than fast IPSPs (Fig. 6*H*)? We observed that fast IPSPs can reach a much more hyperpolarized *V*_M_ without terminating plateaus than slow IPSPs (for which the just-subthreshold trace is shown; Fig. 6*H*, dotted line). As described above, IPSPs hyperpolarize *V*_M_ which rebalances the currents. Because VGCC m-gate updating lags (and particularly so in that *V*_M_ range; Fig. S6*G*), m remains high despite the hyperpolarized *V*_M_, and *I*_VGCC_ increases (Fig. 6*I*). This rebalancing adds to the inward current that the IPSC must overcome to terminate the plateau. Slow IPSPs do not experience this added current load, because m evolves with the IPSP (Fig. 6*I*). This interaction generates a dynamic threshold for plateau termination that collapses when approached with a fast *V*_M_ trajectory (Fig. 6*J*). Overall, these results provide a mechanistic explanation for why slow and sustained inhibition, such as that provided by OLM^Ndnf^, favors plateau termination.

### Dendritic inhibition terminates plateaus evoked under cholinergic influence

Our study so far focused on plateau potentials evoked using intracellular Cs^+^-based solution, which facilitates plateau induction but also alters multiple K^+^ conductances. Long-lasting plateau potentials are readily observed with K^+^-based intracellular solution in vivo (Epsztein et al., 2011; Bittner et al., 2015; Bittner et al., 2017) and in slices under cholinergic influence (Fraser and MacVicar, 1996).

**Figure 7.**
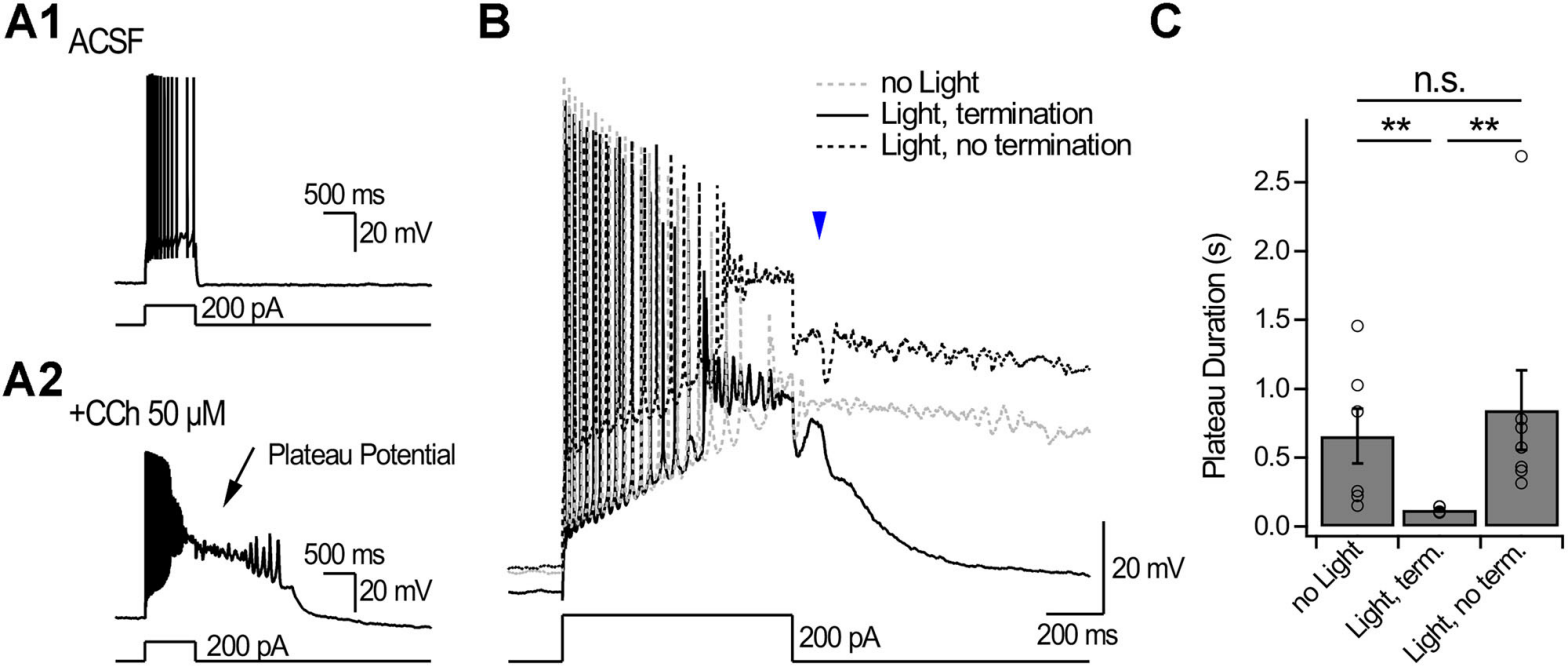
Dendritic inhibition terminates plateaus recorded with K^+^-based intracellular solution under cholinergic modulation. ***A1***, Injection of depolarizing current in a CA1-PYR recorded with K^+^-based intracellular solution reveals firing confined to the current step. ***A2***, Bath application of CCh transformed the response to the same current injection, revealing plateaus that extended beyond the current step. ***B***, Plateaus evoked in presence of CCh can be terminated all-or-none by brief optogenetic stimulation (2 ms) of OLM^Ndnf^ or OLM^α2^. ***C***, Quantification of plateau duration for control trials (no light), trials with optogenetic termination (*n* = 2 neurons in *Ndnf-FlpO;;Nkx2-1-Cre;;Ai80* mice and *n* = 3 neurons in *Chrna2-Cre;;Sst-FlpO;;Ai80* mice), and trials with optogenetic stimulation that did not terminate the plateau (*n* = 1 neuron in *Ndnf-FlpO;;Nkx2-1-Cre;;Ai80* mice and *n* = 6 neurons in *Chrna2-Cre;;Sst-FlpO;;Ai80* mice). Points represent individual neurons. Bars show average ± SEM. n.s. = nonsignificant; **p < 0.01.

We next tested if dendritic inhibition could terminate cholinergic-dependent plateaus recorded with K^+^-based intracellular solution. In ACSF, depolarizing current injection (800 ms, 200 pA) evoked APs limited to the duration of the current step (Fig. 7*A*1). Addition of CCh (50 µM; Fig. 7*A*2) transformed the response to the same current injection steps and revealed plateaus of long duration (658 ± 198 ms; *n* = 6; Fig. 7*A*–*C*). Optogenetic stimulation of OLM^Ndnf^ or OLM^α2^ delivered 100 ms after the end of the depolarizing step could terminate plateaus (likelihood of termination, 41.7%; *n* = 5/12), which significantly shortened their duration (duration, 120 ± 9 ms; *p* < 0.01; Mann–Whitney *U* test, *n* = 5; Fig. 7*B*,*C*). As observed with Cs^+^-based intracellular solution, dendritic inhibition terminated plateaus in an all-or-none manner. In trials where plateaus were not terminated, optogenetic stimulation evoked an IPSP waveform, and plateaus continued their progression such that their duration (845 ± 291 ms; *n* = 7) was much longer than terminated plateaus (*p* < 0.01; Mann–Whitney *U* test; Fig. 7*B*,*C*) but comparable to control trials without light (*p* = 0.73; Mann–Whitney *U* test; Fig. 7*B*,*C*). These results show that plateaus evoked under cholinergic modulation and recorded with a K^+^-based intracellular solution can be terminated by synaptic inhibition, with dynamics similar to plateaus recorded with Cs^+^-based intracellular.

### Plateau termination transforms binary events into graded dendritic Ca^2+^ signals

Large dendritic Ca^2+^ influxes are associated with plateaus, including events supporting BTSP (Cai et al., 2004; Oda et al., 2014; Bittner et al., 2015; Sumegi et al., 2025). We next evaluated the impact of plateau termination on dendritic Ca^2+^ elevations.

**Figure 8.**
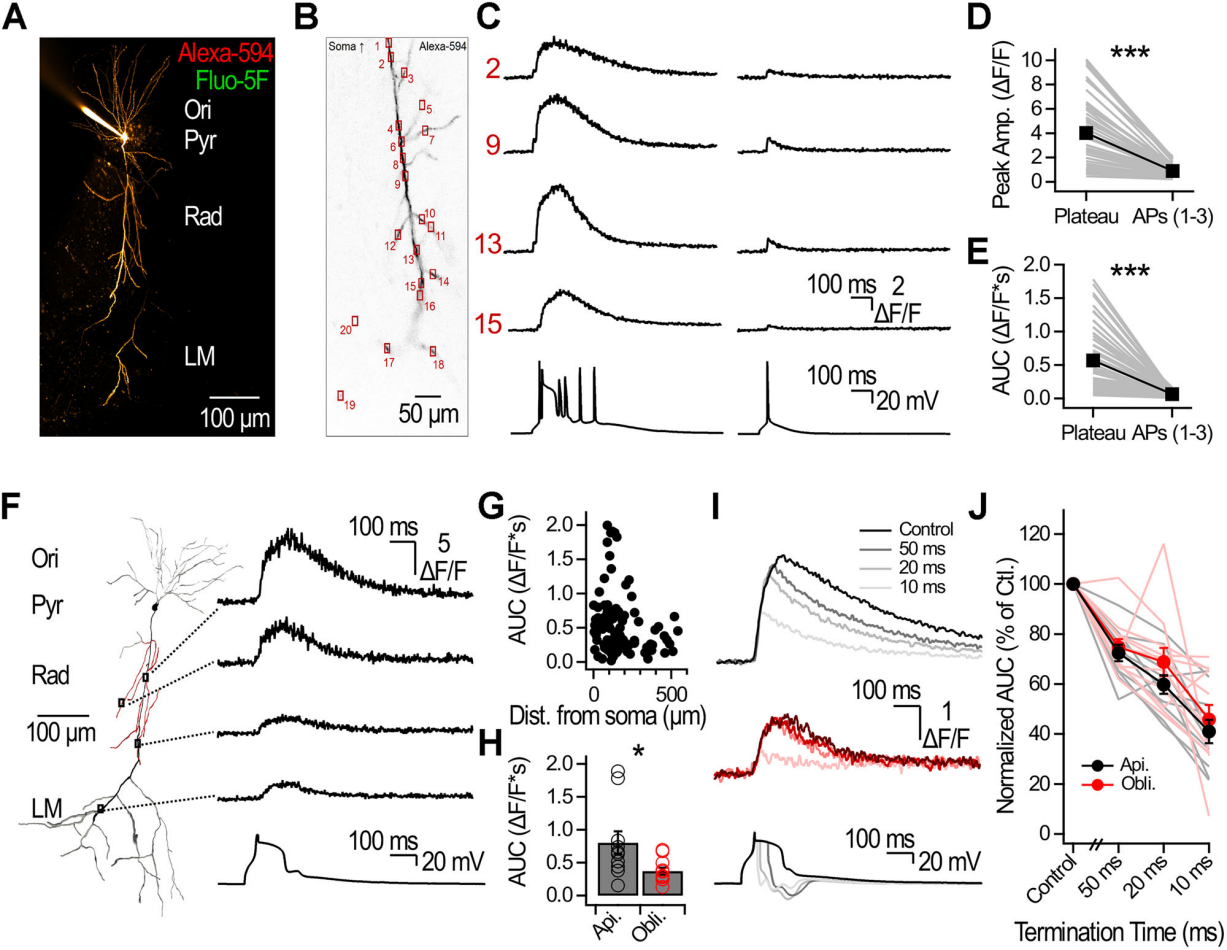
Plateau termination shapes dendritic Ca^2+^ transients. ***A***, Montage from maximal projections of two-photon *Z*-stacks of a CA1-PYR filled with Alexa Fluor 594. ***B***, All recording sites selected on the dendritic arbor for the examples presented in ***C***. ***C***, Dendritic Ca^2+^ transients evoked by AP and plateaus or by single backpropagating AP. The imaging speed was 555 Hz. Numbers correspond to the physical positions shown in ***B***, and all recording sites for that example are shown in Figure S7. Ca^2+^ transients amplitude (***D***) and AUC (***E***) evoked by APs and plateaus or by 1–3 APs without plateaus. Individual lines correspond to a dendritic recording site. Average ± SEM is shown in black. ***F***, Plateau-associated Ca^2+^ transients evoked in the presence of TTX are observed throughout the dendritic arbor. ***G***, Plateau-evoked Ca^2+^ transients recorded in the presence of TTX as a function of distance from the soma. ***H***, Plateau-evoked Ca^2+^ transients in the presence of TTX measured in apical and in obliques dendrites. ***I***, Examples of Ca^2+^ transients recorded in apical (black) and in oblique (red) dendrites associated with control plateaus and at indicated termination times. ***J***, Normalized Ca^2+^ transient AUC as a function of plateau termination time. Data were normalized to the control plateau.

We used random-access two–photon Ca^2+^ imaging to visualize Ca^2+^ elevations in CA1-PYR dendritic arbors. CA1-PYRs were filled with a morphological indicator (Alexa Fluor 594, 40 µM) and the medium-affinity Ca^2+^ indicator Fluo-5F (385 µM; Fig. 8*A*–*C*; Fig. S6; see Materials and Methods for description of the recording sites). Plateaus generated Ca^2+^ transients (CaTs) that were larger and lengthier than CaTs evoked by backpropagating APs (plateau, 4 ± 0.4 Δ*F*/*F*; APs, 0.9 ± 0.1 Δ*F*/*F*; *n* = 57 dendritic sites across *N* = 4 neurons; *p* < 0.001; Wilcoxon signed-rank test; Fig. 8*C*,*D*), yielding a significantly larger area under the curve (AUC) for plateau-evoked CaTs (plateau, 0.57 ± 0.06 Δ*F*/F*s; Aps, 0.06 ± 0.01 Δ*F*/F*s; *n* = 57 dendritic sites across *N* = 4 neurons; *p* < 0.001, Wilcoxon signed-rank test; Fig. 8*E*). Plateaus were isolated from APs with TTX, in which case plateaus still evoked large CaTs across the dendritic arbor (Fig. 8*F*). The duration of the Ca^2+^ transients generally mirrored that of the plateaus recorded electrophysiologically, consistent with maintenance of VGCCs in the activated state (Fig. 8*F*). Plateau-evoked CaTs were observed at long-distances from the soma in primary and secondary branches in the presence of TTX (*n* = 106 dendritic sites across *N* = 6 neurons; Fig. 8*G*).

We focused the analysis on apical and oblique dendrites within the stratum radiatum, which are the primary target sites of Schaffer collateral inputs. Plateau-evoked CaTs were larger in apical dendrites than in the obliques (apical, 0.8 ± 0.17 Δ*F*/F*s; *n* = 10 segments across *N* = 6 neurons; oblique, 0.37 ± 0.05 Δ*F*/F*s; *n* = 11 segments across *N* = 6 neurons; *p* < 0.05; Mann–Whitney *U* test; Fig. 8*H*). Lastly, we probed the impact of plateau termination on dendritic CaTs (Fig. 8*I*). Plateaus were terminated with mock IPSPs at 10, 20, and 50 ms, and the AUC of plateau-evoked CaTs was compared with control nonterminated plateaus in paired measurements (Fig. 8*I*,*J*). Plateau termination diminished CaTs relative to control such that shorter plateaus gave rise to smaller CaTs in apical and oblique dendrites (apical, *n* = 10 segments; oblique, *n* = 11 segments; both recorded across *N* = 6 neurons; Fig. 8*J*). Therefore, these results show that plateau termination effectively grades dendritic CaTs.

## Discussion

Our results show that plateaus recorded in CA1-PYRs are terminated in an all-or-none manner by dendritic inhibition. Plateau duration is determined by intrinsic CA1-PYR properties and the feedback inhibitory circuit. Plateau termination controls dendritic Ca^2+^ signals critical for synaptic plasticity. Because both plateaus and feedback inhibition are implicated in plasticity and memory (Mehta et al., 2002; Leao et al., 2012; Lovett-Barron et al., 2014; Bittner et al., 2017; McKenzie, 2018; Siwani et al., 2018; Fernandez-Arroyo et al., 2025; Bott et al., 2025; Udakis et al., 2025), we consider the consequences of plateau termination for circuit dynamics and branch-specific dendritic processing.

Plateau termination may promote sparse ensemble formation by limiting Ca^2+^-dependent plasticity in select neurons. The local inhibitory circuit organizes CA1-PYRs into functional ensembles for spatial representation, and feedback inhibition constrains ensemble size (Grienberger et al., 2017; Geiller et al., 2022; Rolotti et al., 2022; Adaikkan et al., 2024). OLM^α2^ are implicated in hippocampal-dependent memory and their activity can be modulated by learning (Siwani et al., 2018; Bott et al., 2025; Udakis et al., 2025; Campbell et al., 2026). Although the roles of OLM^Ndnf^ remain less well defined, OLM subtypes may be differentially recruited across behavioral states via subtype-specific synaptic circuits (Johantges et al., 2025).

Global suppression of feedback inhibition enhances local population spiking and increases the amplitude and duration of ripples (Adaikkan et al., 2024). Sharp-wave ripples (SPW-Rs) are high-frequency oscillations that are important for memory and involve inhibitory interactions (Stark et al., 2014; Buzsaki, 2015; Fernandez-Ruiz et al., 2019). The co-occurrence of complex Ca^2+^ spikes in CA1-PYR dendrites with SPW-Rs supports the idea that independent representations may be achieved through the combination of neuron-specific plateau termination thresholds and inhibitory connectivity motifs (Buzsaki et al., 1996; Kamondi et al., 1998; Roux and Buzsaki, 2015).

We further hypothesize that local plateau terminations spatially confine Ca^2+^ elevations to constrain synaptic plasticity in select branches (Leao et al., 2012; Bloss et al., 2016; Udakis et al., 2025). Individual CA1-PYRs can support multiple spatial representations, likely through dendritic compartmentalization that enables branch-specific encoding (Mehta, 2004; Leutgeb et al., 2005; Bittner et al., 2015; Sheffield and Dombeck, 2015; O'Hare et al., 2025). Plateau compartmentalization allows individual neurons to detect input sequences over behaviorally relevant timescales (Leugering et al., 2023). Plateaus evoked and recorded in single CA1-PYR dendritic branches can exceed 100 ms and spread to neighboring branches (Wei et al., 2001; Cai et al., 2004). Dendritic inhibition possibly guides local plastic changes to establish sparse and selective representations within a CA1-PYR neuron (Mehta, 2015; Grienberger et al., 2017).

### Reconstruction of plateau termination

This study adds to the growing body of evidence that dendritic spikes and plateaus are highly sensitive to inhibition (Callaway et al, 1995; Kim et al., 1995; Miles et al., 1996; Bloss et al., 2016; Doron et al., 2017; Du et al., 2017). In CA1-PYRs, our electrophysiological recordings, Ca^2+^ imaging, and computational modeling collectively demonstrate that plateaus arise from VGCC activation, which generates a Ca^2+^ current that sustains membrane depolarization through a positive feedback loop. Ca^2+^ activates I_SK_, forming a system where two currents of opposing polarity nearly balance each other (Wei et al., 2001; Cai et al., 2004). The small net outward current slowly repolarizes *V*_M_ until a threshold for rapid repolarization is reached, given by the point where the inward current can no longer sustain the positive feedback loop, and VGCC deactivation abruptly repolarizes *V*_M_ back to the resting state. Synaptic inhibition adds to the outward current pressure and forces plateau termination.

Gradually evolving stable and threshold points explain all-or-none plateau repolarization in CA1-PYRs, in a similar manner to cardiac Purkinje fibers and neocortical pyramidal cells (Weidmann, 1951; Noble and Tsien, 1969; Reuveni et al., 1993). Our results further show that the dynamic evolution of the threshold on two timescales governs the impact of synaptic inhibition. The threshold approximated under steady-state conditions shifts toward a more depolarized value during the plateau, conferring an initial resistance to inhibition that gradually dissipates. For a subthreshold IPSP, *V*_M_ hyperpolarization increases the driving force for Ca^2+^ and increases the current which repolarizes *V*_M_ back to the plateau phase. This is because VGCC m-gate updating is particularly slow in that *V*_M_ range and lags rapid hyperpolarization (Fig. S6*G*). The interaction generates a dynamic threshold that collapses when approached with an elevated dV/dt. This is conceptually the inverse of AP generation, where slow depolarization can fail to trigger firing due to accommodation (Hodgkin and Huxley, 1952). Termination threshold is not a fixed *V*_M_, but rather a dynamically evolving point governed by prior activity and intracellular Ca^2+^ dynamics. This explains why slow IPSPs mediated by OLM^Ndnf^ more effectively terminate plateaus than the faster IPSPs generated by OLM^α2^. It is anticipated that plateau termination by synaptic inhibition is broadly applicable across systems and species, given the prevalence of VGCCs in mediating multiple forms of dendritic plateaus (Reuveni et al., 1993; Larkum et al., 2022).

### Intrinsic mechanisms supporting plateaus

Plateaus in this study were of long duration but well within the range of plateaus recorded in vivo with K^+^ internal solution (Epsztein et al., 2011; Bittner et al., 2015; Bittner et al., 2017). Plateaus in acute slices are made evident by using Cs^+^-based intracellular solution (Bittner et al., 2017), cholinergic modulation (Fraser and MacVicar, 1996), coincident excitatory inputs activation (Takahashi and Magee, 2009), dendritic glutamate uncaging (Wei et al., 2001; Cai et al., 2004; Yang et al., 2014), and high activity levels (Suzuki et al., 2008). In the present study, Cs^+^-based intracellular solution was used to facilitate reliable plateau generation and allow precise temporal control of inhibitory input relative to ongoing plateaus. Importantly, we observed similar all-or-none termination dynamics for plateaus recorded with K^+^-based intracellular solution under cholinergic modulation. The synaptic dynamics providing the depolarization required for plateau initiation are important to understand dendritic computations, but this aspect is not the focus here (Takahashi and Magee, 2009; Bittner et al., 2017; Xiao et al., 2023; Jain et al., 2024). Future studies will reveal how inhibition shapes plateaus recorded in vivo under unperturbed conditions.

Our single-compartment model captures a phenomenon acting across the complex CA1-PYR dendritic arbor that contains multiple types of heterogeneously distributed channels (Hell et al., 1993; Poirazi et al., 2003a,b). While the model explains the main determinants of plateau termination, differences between experiments and model remain. We and others previously observed that plateaus end spontaneously in the presence of apamin, are shortened by TTX, and can be regenerated. Our results further show that IPSPs become larger when delivered later during plateaus despite constant optogenetic stimulation, a feature not captured by the model. Additional mechanisms and conductances not accounted for in our model likely contribute to support these features (Colling and Wheal, 1994; Fraser and MacVicar, 1996; Golding et al., 1999; Magee and Carruth, 1999; Robinson and Siegelbaum, 2003; Cai et al., 2004; Tsay et al., 2007; Hansen et al., 2014; Combe et al., 2023). Our latter observation requires closer attention, as it could enhance the plateaus' early resistance to synaptic inhibition. A Ca^2+^-activated Cl^−^ current contributes to AP repolarization in CA1-PYRs and is likely to be amplified by the larger Ca^2+^ elevations generated by plateaus (Huang et al., 2012). Decaying intracellular Cl^−^ concentration during the plateau provides an attractive explanation for the growing IPSP amplitude not associated with changes in input resistance. Depending on the distribution of this current across the dendritic arbor, it may confer resistance to inhibition in select compartments or even contribute to early plateau termination.

### Structure, function, and timing of specialized feedback inhibitory elements

The timing of inhibition determines whether plateaus are terminated or allowed to persist. Repetitive CA1-PYR spiking optimally recruit INs including OLM^Ndnf^ and OLM^α2^ (Ali and Thomson, 1998; Losonczy et al., 2002; Pouille and Scanziani, 2004; Sylwestrak and Ghosh, 2012; Chamberland et al., 2024). Delayed postintegration recruitment is aligned with the role of *Sst*-INs in regulating CA1-PYR activity toward the end of place fields or during burst firing (Royer et al., 2012). Previous work reported that optogenetic activation of *Sst*-INs produced only modest reductions in the dendritic trunk spike area (Milstein et al., 2015). The more pronounced effects observed here likely reflect the importance of the temporal relationship between inhibition and the plateau. Plateaus themselves can provide the signal for a CA1-PYR to recruit feedback inhibition by triggering axonal spikes (Apostolides et al., 2016). OLMs receive over 5,000 excitatory synapses, mostly from local CA1 collaterals which usually form a single contact site per cell (Gulyas et al., 1993; Blasco-Ibanez and Freund, 1995; Booker and Vida, 2018; Takacs et al., 2024). Therefore, multiple modes of activity in the CA1 circuit are expected to recruit OLMs.

Our experiments and modeling results converge to show why slower inhibitory currents drive plateau termination more effectively, providing a distinct advantage to OLM^Ndnf^ over OLM^α2^ for terminating plateaus with more efficacy or at earlier timepoints. The importance of OLM^Ndnf^ for plateau termination and inhibitory control of CA1-PYRs is further amplified by our recent demonstration that OLM^Ndnf^ preferentially target CA1-PYRs over fast-spiking INs, whereas OLM^α2^ strongly innervate both targets (Chamberland et al., 2024). While OLM^Ndnf^ and OLM^α2^ are transcriptomically similar and exhibit a common anatomical phenotype (Harris et al., 2018; Chamberland et al., 2024), our data demonstrated only minimal overlap between the two subpopulations in the transgenic animals used. Both anatomical datasets show that OLM^Ndnf^ and OLM^α2^ exhibit variable axonal arborization within stratum oriens, with a greater proportion of OLM^α2^ cells exhibiting local collaterals in the present dataset. The complete circuit wiring across hippocampal layers remains to be determined for both subtypes. Anatomical analysis confirmed our previous observations that OLM^Ndnf^ axons penetrate LM less than those of OLM^α2^, arguing that dendritic filtering is unlikely to account for the differences in IPSC kinetics (Chamberland et al., 2024). Slower IPSCs mediated by OLM^Ndnf^ may arise from differences in presynaptic release properties and/or postsynaptic receptor composition, which could include variation in GABA_A_R subunits or recruitment of slower inhibitory conductances, although the precise molecular mechanisms remain to be determined. This observation, combined with the fact that plateau termination is a cell-intrinsic mechanism, supports the general principle that any sufficiently strong and slow inhibition can trigger terminations to control dendritic Ca^2+^ elevations.

### Biophysical model code for peer review

The original code for the biophysical model can be found here: https://modeldb.science/2019872 (password: Termination2025).
